# Genome Sequence of *Streptococcus agalactiae* Strain 09mas018883, Isolated from a Swedish Cow

**DOI:** 10.1128/genomeA.00456-13

**Published:** 2013-07-11

**Authors:** S. Zubair, E. P. de Villiers, H. H. Fuxelius, G. Andersson, K.-E. Johansson, R. P. Bishop, E. Bongcam-Rudloff

**Affiliations:** Department of Animal Breeding and Genetics, SLU Global Bioinformatics Centre, Swedish University of Agricultural Sciences, Uppsala, Swedena; International Livestock Research Institute, Nairobi, Kenyab; Department of Biomedical Sciences and Veterinary Public Health, Swedish University of Agricultural Sciences, Uppsala, Swedenc

## Abstract

We announce the complete genome sequence of *Streptococcus agalactiae* strain 09mas018883, isolated from the milk of a cow with clinical mastitis. The availability of this genome may allow identification of candidate genes, leading to discovery of antigens that might form the basis for development of a vaccine as an alternative means of mastitis control.

## GENOME ANNOUNCEMENT

*Streptococcus agalactiae*, also known as group B streptococcus (GBS), is a Gram-positive pathogen causing sepsis, meningitis, and pneumonia in human neonates (1) and subclinical mastitis in dairy cattle (2). It infects heifers that have not yet calved and also older cattle in dairy herds in the absence of effective control programs. It is one of the leading causes of economic losses in the dairy industry (3, 4). We have determined the complete genome sequence of an *S. agalactiae* strain, 09mas018883, isolated from bovine (*Bos taurus*) milk.

The genome of *S. agalactiae* 09mas018883 was sequenced by using an Illumina GAIIx employing a paired-end read library strategy with a mean library insert size of 545 bp. After filtering of low-quality reads, MIRA v3.0.0 (5) was used to assemble a total of 10,079,600 quality reads with an average length of 75 bp. The approach of comparative mapping combined with *de novo* assembly was used, followed by gap closure by PCR and Sanger sequencing, GapFiller (6), and Velvet *de novo* assembly (7), ultimately aligning 10,035,130 reads (99.56%) to the genome with an average coverage of 351×. Whole-genome annotation was performed with BASys (8) and RAST (9), and the genome sequence was further analyzed with Artemis and the Artemis Comparison Tool (10).

*S. agalactiae* 09mas018883 has a circular chromosome of 2,138,694 bp with a G+C content of 35.55%. BASys predicted 2,081 protein-coding genes (CDSs). The open reading frames (ORFs) with putative assigned functions comprise approximately 70%. A total of 80 tRNA genes were predicted by tRNAscan-SE 1.21 (11). rRNA genes were identified by using RNAmmer (12). A total of 21 rRNA genes were predicted, with 7 copies of 16S, 23S, and 5S rRNA genes each.

Genomic islands are the regions of a genomic sequence that vary among closely related strains. These are known to have an important role in the evolution of bacteria by conjugation, transduction, transformation, or horizontal gene transfer, thus underpinning the adaptive capacity of bacteria (13). Genomic islands were identified using IslandViewer (14). Strain 09mas018883 has 7 predicted genomic islands containing 88 genes, including insertion elements, phage genes, prophage genes, membrane-associated proteins, and gene clusters, such as the *purDEK* and *cyl* operons. The *purDEK* operon encodes enzymes that are important for energy metabolism in the *de novo* purine biosynthetic pathway, thus aiding bacterial growth in milk (15). Pilus-like structures are important virulence factors. Genes encoding candidate virulence factors are located in two genomic islands, which each contain at least three genes encoding proteins that contain the conserved amino acid motif LPXTG (16). The 09mas018883 genome contains two type 1 pathogenicity islands, PI-1 and PI-2A. Strain 09mas018883 possesses capsular polysaccharide genes that are also important potential virulence factors. There is an insertion sequence, 2,772 bp in length, containing three predicted genes between the *cpsG* and *cpsH* genes within the *cps* region. The *cps* antigens are also used for the serotyping of GBS isolates (17). The comparative analysis and annotation of the genome are in progress and will be documented in greater detail in a future publication.

### Nucleotide sequence accession number.

The complete genome sequence of *S. agalactiae* strain 09mas018883 has been deposited in the European Nucleotide Archive (ENA) under the accession no. HF952104.
